# Improved fat water separation with water selective inversion pulse for inversion recovery-based cardiac MRI sequence

**DOI:** 10.1186/1532-429X-14-S1-P276

**Published:** 2012-02-01

**Authors:** Lukas Havla, Tamer A Basha, Hussein Rayatzadeh, Jaime L Shaw, Warren J Manning, Scott B Reeder, Sebastian Kozerke, Reza Nezafat

**Affiliations:** 1 Department of Medicine, Harvard Medical School and Beth Israel Deaconess Medical Center, Boston, MA, USA; 2 Department of Radiology, Harvard Medical School and Beth Israel Deaconess Medical Center, Boston, MA, USA; 3 Institute for Biomedical Engineering, University and ETH Zurich, Zurich, Switzerland; 4 Department of Radiology, University of Wisconsin, Madison, WI, USA

## Background

Chemical shift based water-fat separation methods can be used to reconstruct simultaneous fat and water images, thereby improving the sensitivity of fat detection using a positive fat contrast. The presence of fatty infiltration is a hallmark pathological feature of arrhythmogenic right ventricular cardiomyopathy (ARVC). Furthermore, the presence of fatty infiltration in chronic myocardial infarction has been demonstrated recently [1,2]. To assess for both presence of scar using late gadolinium enhancement (LGE) and fat, two separate scans are needed. In this study, we propose an improved inversion recovery based water-fat separation sequence in which the fat signal is retained by the application of a spectrally selective water inversion pulse, thereby eliminating the need for two separate scans.

## Methods

A free-breathing 3D LGE sequence with multi-echo gradient echo imaging sequence and a water-selective inversion pulse was implemented on 1.5T Philips Achieva system. To evaluate the efficacy of the proposed sequence, imaging in phantom were performed. The phantom contains multiple vials with different T1s and a vegetable oil vial. Subsequently, fat-water separation was performed in 8 patients referred for evaluation of ARVC. The 3-echo 3D GRE sequences are respiratory navigator-gated (acceptance windows 5mm) and ECG-triggered with in-plane spatial resolution of 1.5x1.2mm2 and slice thickness 2.0 or 4.0mm. TR/TE1/TE2/TE3/α= 8.0/1.5/4.0/6.5/15°, FOV= 300x300x100mm3. The IDEAL with a region growing technique [3,4] was used to reconstruct separate water and fat images.

## Results

**Figure 1:**
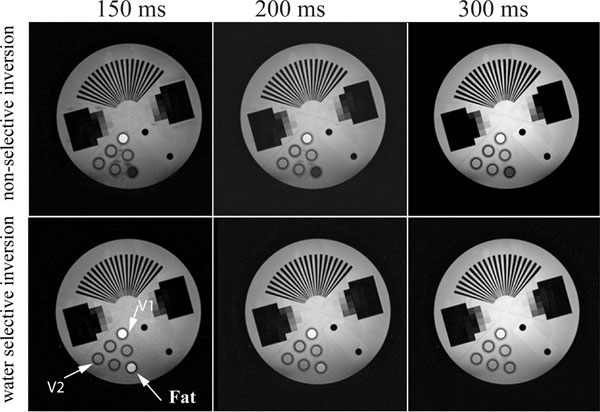
Phantom images show retained fat signal (bottom row) opposed to non-selective inversion pulse (top row) over different inversion times TI (columns). V1 and V2 contain Gd-dopped water solution.

**Figure 2:**
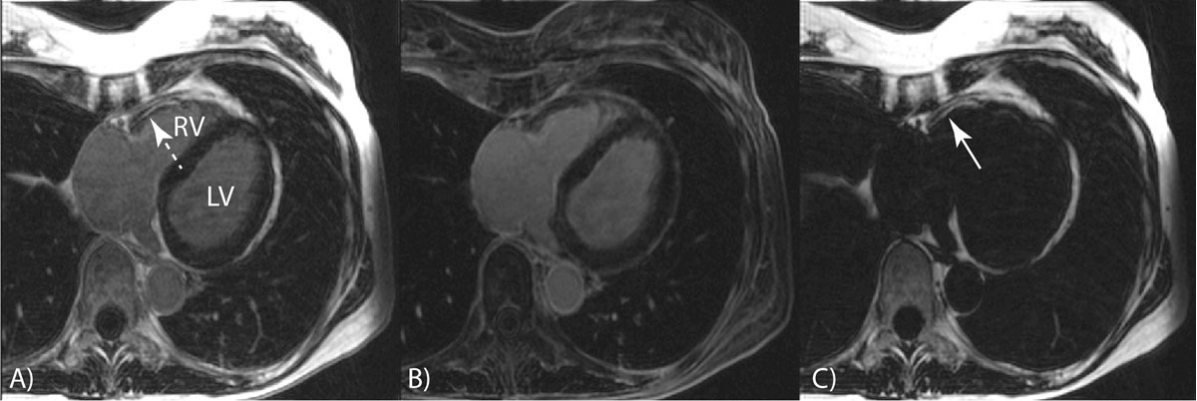
(A) first echo, (B) water and (C) fat images from a suspected ARVC patient acquired using a water-selective IR. The presence of fatty infiltration in the RV free wall can be easily visualized. The fat signal can also be visualized in the first-echo images prior to water-fat separation.

Figure 1 shows a representative slice from the phantom experiment, comparing images acquired using non-selective (top row) and water selective inversion (bottom row) with different inversion times of 150 ms, 200 ms and 300 ms. The fat signal is significantly decreased in images acquired with non-selective inversion; however, the use of a water-selective inversion retains fat signal allowing robust water-fat separation. Figure 2 shows a slice from 3D dataset showing a) first echo, b) water and C) fat images from a suspected ARVC patient acquired using a water-selective IR. The presence of fatty infiltration in the RV free wall can be easily visualized in the water and fat images. However there is no evidence of enhancement in LGE. This was confirmed in a subsequent scan using standard single-echo LGE with non-selective inversion.

## Conclusions

The water-selective inversion pulse significantly improves visualization of the fat signal in inversion-recovery based water-fat separation, thereby allowing the assessment of water and fat content and fibrosis/scar using LGE in a single scan.

## Funding

The authors acknowledge grant support from NIH R01EB008743-01A2. Lukas Havla was supported by a fellowship from Bayer Science & Education Foundation.
